# High‐density genotyping of the A.E. Watkins Collection of hexaploid landraces identifies a large molecular diversity compared to elite bread wheat

**DOI:** 10.1111/pbi.12757

**Published:** 2017-07-28

**Authors:** Mark O. Winfield, Alexandra M. Allen, Paul A. Wilkinson, Amanda J. Burridge, Gary L.A. Barker, Jane Coghill, Christy Waterfall, Luzie U. Wingen, Simon Griffiths, Keith J. Edwards

**Affiliations:** ^1^ University of Bristol Bristol Life Sciences Building 24 Tyndall Avenue Bristol UK; ^2^ John Innes Centre Norwich Research Park Norwich Norfolk UK

**Keywords:** landraces, SNPs, *Triticum aestivum*, Watkins Collection, wheat

## Abstract

The importance of wheat as a food crop makes it a major target for agricultural improvements. As one of the most widely grown cereal grains, together with maize and rice, wheat is the leading provider of calories in the global diet, constituting 29% of global cereal production in 2015. In the last few decades, however, yields have plateaued, suggesting that the green revolution, at least for wheat, might have run its course and that new sources of genetic variation are urgently required. The overall aim of our work was to identify novel variation that may then be used to enable the breeding process. As landraces are a potential source of such diversity, here we have characterized the A.E. Watkins Collection alongside a collection of elite accessions using two complementary high‐density and high‐throughput genotyping platforms. While our results show the importance of using the appropriate SNP collection to compare diverse accessions, they also show that the Watkins Collection contains a substantial amount of novel genetic diversity which has either not been captured in current breeding programmes or which has been lost through previous selection pressures. As a consequence of our analysis, we have identified a number of accessions which carry an array of novel alleles along with a number of interesting chromosome rearrangements which confirm the variable nature of the wheat genome.

## Introduction

Bread wheat (*Triticum aestivum*), with production of more than 730 million tonnes in 2015 constituting 29% of global cereal production, is the main provider of calories in the diet globally (FAO Cereal Supply and Demand Brief). However, while global demand for wheat is increasing by 1.7% each year due to population growth and increase in average incomes (Lobell *et al*., 2009), wheat yields have plateaued over the last 10–15 years (Grassini *et al*., 2013); with a projected decline in wheat productivity over the coming years of between 6 and 8% (up to 25% for some tropical regions) due to climate change (Schleussner *et al*., 2016), it seems that wheat production is not on track to meet demand. Thus, wheat breeders are faced with a series of challenges: increase genetic gains in productivity at a rate not lower than growing demand; increase grain yield and yield stability; increase resistance/tolerance to biotic and abiotic stresses; improve end‐use and nutritional quality characteristics.

Modern breeding and monoculture cropping have greatly improved yield and quality, but might have resulted in a reduction of genetic variation potentially making crops more vulnerable to disease and climate change. It is suggested that, during the ‘Green Revolution’, breeding programmes were based on a small number of target genes (e.g. *Rht* dwarfing genes in wheat) such that elite lines experienced a bottle neck that reduced genetic diversity (Doebley *et al*., 2006; Khush, 2001; Roussel *et al*., 2005). This might not generally be the case, however, since breeding programmes in different countries and at different times have produced variable results with respect to preserving underlying levels of genetic diversity (van de Wouw *et al*., 2009). For instance, while some authors have reported a decrease in genetic diversity (Cavanagh *et al*., 2013; Roussel *et al*., 2004) others suggest that there has been no loss of genetic diversity (CIMMYT 2014 Annual report) or that there has been a qualitative rather than a quantitative shift in genetic diversity (Donini *et al*., 2000). Whatever the reality, novel sources of genetic variability are required to allow breeders to face the challenge of increasing wheat yields in an insecure future.

Collections of old landraces may hold novel variability not present in modern elite lines (Lopes *et al*., 2015; Riaz *et al*., 2016; Vikram *et al*., 2016). As long ago as the 1980s, it was recognized that old, bread wheat landrace populations represent an important reservoir of genetic diversity that can be drawn upon for the improvement of modern elite varieties (Feldman and Sears, 1981). Landraces have been shown to be a potential source of resistance to both drought (Reynolds *et al*., 2007) and disease (Bansal *et al*., 2011, 2013; Burt *et al*., 2014; Toor *et al*., 2013). They may also harbour valuable variation for traits associated with good agronomic performance and quality. For example, the Portugese landrace ‘Barbela’ shows notable variation for the wheat storage proteins high and low molecular weight gliadins (HMW and LMW‐GS) (Ribeiro‐Carvalho *et al*., 2004). However, breeding with landraces, which may carry desirable genes but which otherwise may not be optimal for agricultural use, can lead to yield penalties caused by the introduction of non‐adapted alleles that are linked to genes of interest. The availability of molecular markers enables breeders to cross in desired genes and then recover the elite genotype, but to do so efficiently requires there to be extensive genome coverage by markers that are highly polymorphic between cultivars. The recent development of various high‐density platforms for wheat genotyping (e.g. LGC's KASP platform, Illumina's iSelect platform and Affymetrix's Axiom platform) has made possible the analysis of such crosses to target desirable introgressions into elite lines.

The Watkins Collection of hexaploid wheats, the main subject of this article, has been identified as a potentially valuable collection of landraces. The accessions in this collection, which were acquired during the 1930s from local markets in 32 countries in Asia, Europe and Africa, represent a snap shot of genetic diversity present before the onset of modern breeding practices. It has been shown that, for many phenotypic characters of agronomic importance, the accessions belonging to the landrace collection are more variable than accessions from a collection of modern European bread wheat varieties (Wingen *et al*., 2014). The collection was also shown to have a much higher level of genetic diversity and has been used to identify novel loci for rust resistance (Bansal *et al*., 2011, 2013; Randhawa *et al*., 2015; Toor *et al*., 2013) and eyespot resistance (Burt *et al*., 2014). However, the genetic analysis of Wingen *et al*. (2014) was carried out using a small number of microsatellite markers (41 microsatellite markers covering all 21 wheat chromosomes). Since that time, a large number of SNP markers and new platforms for high throughput analysis have been developed that give genome‐wide coverage (Allen *et al*., 2016; Winfield *et al*., 2016). Hence, one of the main aims of this study was to use these new markers and platforms to see whether similar conclusions would be drawn with regard to the Watkins Collection of hexaploid lines and, in addition, identify novel markers that might be unique to this Collection and, hence, not found in elite accessions. From a biological perspective, such information could be used by breeders and academics to inform future breeding strategies with a view to increasing the extent of genetic variation present within the elite accession gene pool. It has been estimated that 10 000–20 000 markers are necessary to have a reasonably high‐density map in bread wheat (Appels, 2003). In this study, we have surpassed this barrier by more than an order of magnitude using an array with more than 800 000 markers (Winfield *et al*., 2016).

## Results

### Geographic Distribution of the genetic variability in the Watkins accessions

**Figure 1:**
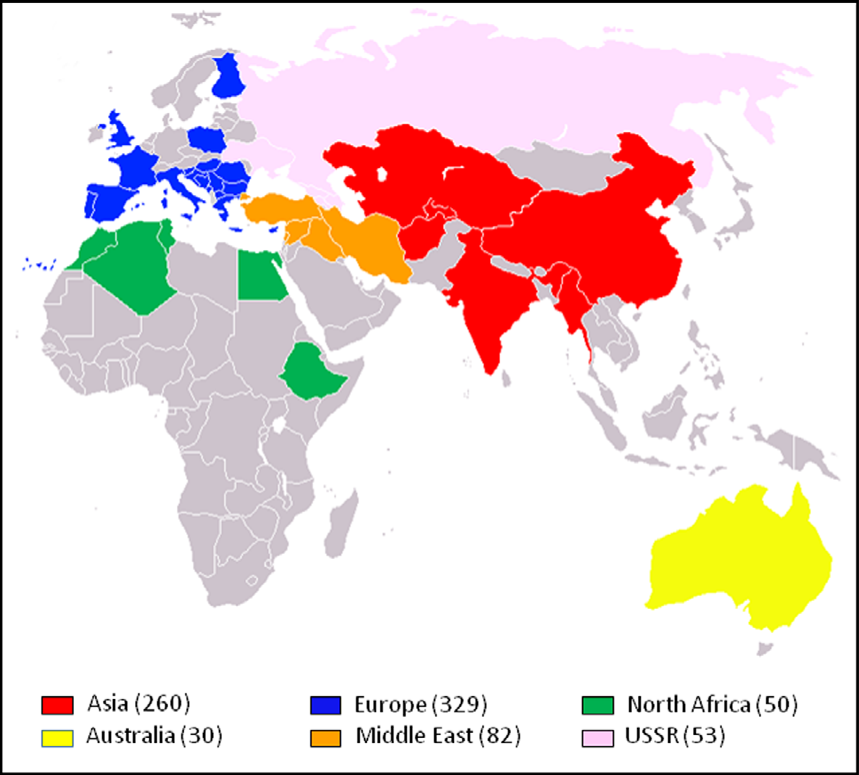
The six broad regions from which the Watkins accessions were collected. The numbers in brackets are the number of accessions from each of the regions. The European accessions include 13 from the Canary Islands. A total of 804 accessions were included in the study.

**Figure 2:**
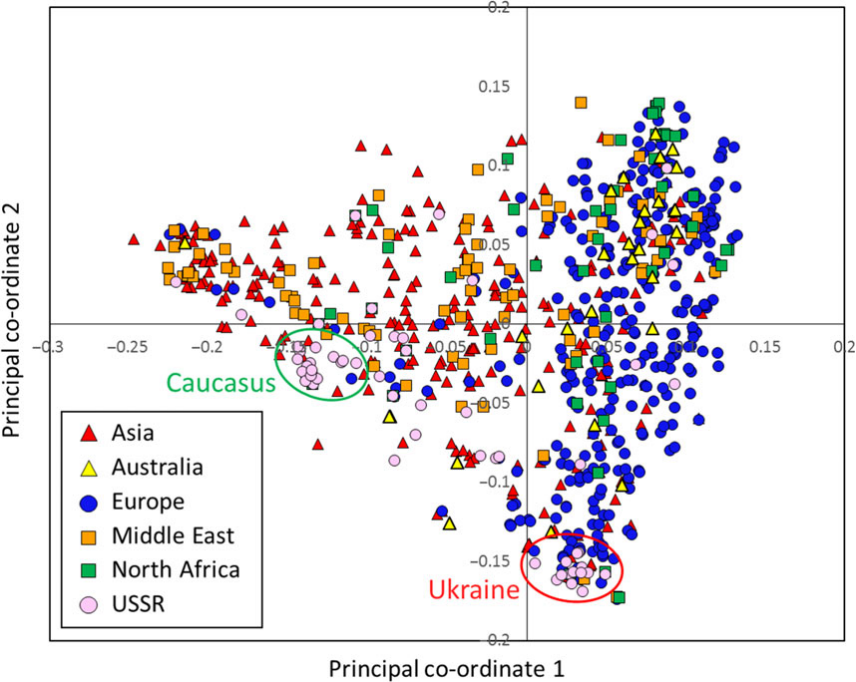
PCO plot coloured by region of origin. The Asian and Middle Eastern accessions are quite evenly distributed across the plot, whereas the European accessions cluster to the right of the plot. Australian and North African accessions predominately cluster with the European accessions. Accessions from the USSR form two distinct clusters; most of the accessions collected in the Caucasus (Armenia, Azerbaijan, Georgia) cluster with Asian and Middle Eastern accessions (green oval); a small group containing the accessions from the Ukraine, Crimea and Eastern Russia cluster with accessions from Eastern Europe (red oval).

The 804 hexaploid wheat accessions of the Watkins Collection used in this study were collected from more than thirty countries. In order to allow comparison between geographic origin and genotype data, the accessions were grouped into six broad geographical regions (Fig. 1 and Table 1). Genotyping was performed using the 35 k Wheat Breeders’ Array, which contains 35 143 SNP probes (Allen *et al*., 2016). For this set of 804 accessions, there were 32 443 polymorphic markers (92.3%% of probes on the array) – only high quality marker as defined in Winfield *et al*. (2016) was included in this study. Population structure within the collection was determined using principal co‐ordinate (PCO) and STRUCTURE analysis (Pritchard *et al*., 2000). At the level of the broad geographical regions, the PCO plot showed loose clustering of the accessions (Fig. 2). Asian and Middle Eastern accessions were quite evenly distributed along the first axis but less evenly distributed along axis two with most Middle Eastern accessions falling in the positive (upper) part of the plot. The accessions from Australia, Europe and North Africa principally clustered towards the right side (positive values) of the first co‐ordinate axis. The second axis explained very little at the regional level. However, when country of origin was taken into account, it splits the accessions from Europe into two broad groups: accessions from the Canary Islands, Crete, Cyprus, France, Greece, Italy, Portugal, Spain and the UK (hereafter referred to as Western European accessions) were principally located in the upper right of the PCO plot; accessions from Bulgaria, Finland, Hungary, Poland, Romania and Yugoslavia (hereafter referred to as Eastern Europe accessions) were principally located in the lower right of the plot. Accession from Australia and North Africa clustered with those of Western Europe. Interestingly, accessions from the former USSR fell into two separate clusters: one co‐localized with the Eastern European accessions, the other clustered with the Asian/Middle Eastern accessions. The Middle Eastern accessions originating in Iran mainly clustered away from the European accessions while those from Turkey clustered with accessions from Western Europe (File S2). The accessions from the North African countries Algeria and Tunisia did not show tight clustering. Those from Morocco, however, tended to cluster with accessions from Western Europe. The USSR accessions from the Ukraine and Eastern Russia (Rivers Don and Volga) clustered with the Eastern European accessions from Poland, Bulgaria and Hungary while those from Armenia, Azerbaijan and Georgia clustered with accessions from the Middle East and Asia (Fig. 2).

**Table 1:** The six broad regions and individual countries from which accessions were collected

Region	Country	No. Acc.
Asia	Afghanistan	35
	Burma (Myanmar)	4
	China	87
	India	128
	Turkestan	6
Australia	Australia	30
Europe	Bulgaria	14
	Canary Islands	13
	Crete	11
	Cyprus	2
	Finland	1
	France	21
	Greece	26
	Hungary	7
	Italy	18
	Poland	20
	Portugal	39
	Romania	7
	Spain	99
	United Kingdom	3
	Yugoslavia	48
Middle East	Iran	52
	Iraq	8
	Palestine	2
	Syria	4
	Turkey	16
North Africa	Algeria	7
	Egypt	4
	Ethiopia	2
	Morocco	21
	Tunisia	16
USSR	USSR	53

**Figure 3:**
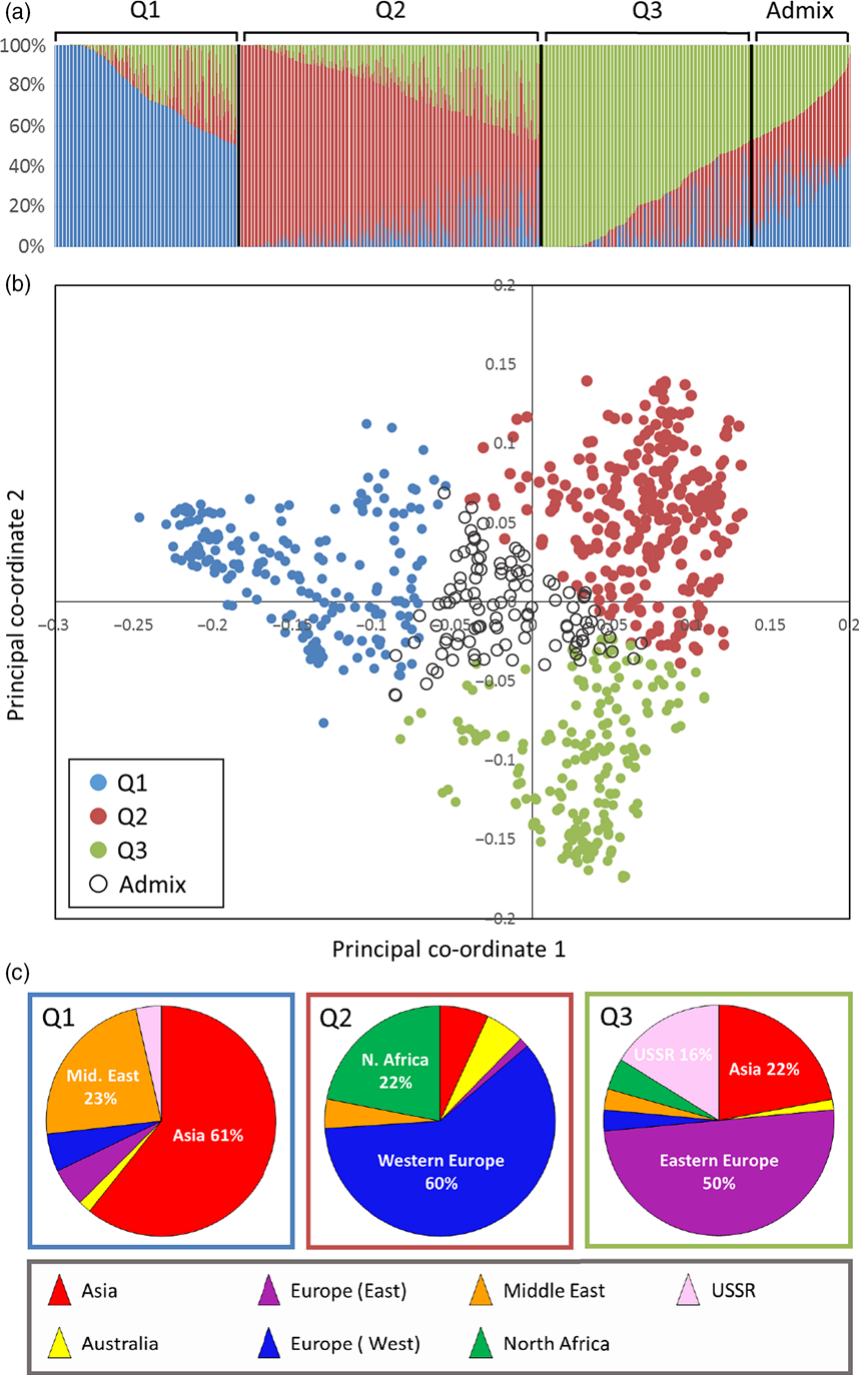
(a) Results of the STRUCTURE analysis indicated that the optimum *K* value was 3. The individual lines were assigned membership coefficients to each of these three clusters (Q1, Q2 and Q3) if they had 50% or greater membership to that group. A forth group, ‘Admix’ contained those lines that had less than 50% apportioned to all three groups. (b) The lines in the PCO plot are coloured according to their assignment to the four groups. (c) The pie charts show the regions from which the accessions, with greater than 90% assignment to one of the groups, were collected.

STRUCTURE analysis indicated *K* = 3 to be the optimum partitioning of the 804 accessions (Fig. 3a). Overlaying the STRUCTURE predictions onto the PCO plots showed that there was good agreement between the two clustering methods (Fig. 3a,b). The first principal co‐ordinate separated individuals in group 1 of the STRUCTURE analysis from those in groups 2 and 3. The second co‐ordinate separated the individuals in groups 2 and 3 of the STRUCTURE analysis. Individual accessions that have 90% or greater membership to any one of the groups form the points of the PCO distribution: the accessions with >90% membership to group 1 were principally from Asia and the Middle East; group 2 was dominated by Western European lines and those from North Africa; group 3 was dominated by lines from Eastern Europe and Asia (Fig. 3c). Thus, STRUCTURE analysis sustained the patterns seen in the PCO plots: it supported the separation of the European accessions into two distinct groups (this is highlighted in Fig. 3c by the inclusion of an additional regional category, ‘Eastern Europe’); it showed the separation of the accessions from the former USSR into two ancestral groups, one being part of the Asian cluster, the other clustering with the accessions of Eastern European origin; it supported the similarity of Western European and North African accessions.

**Figure 4:**
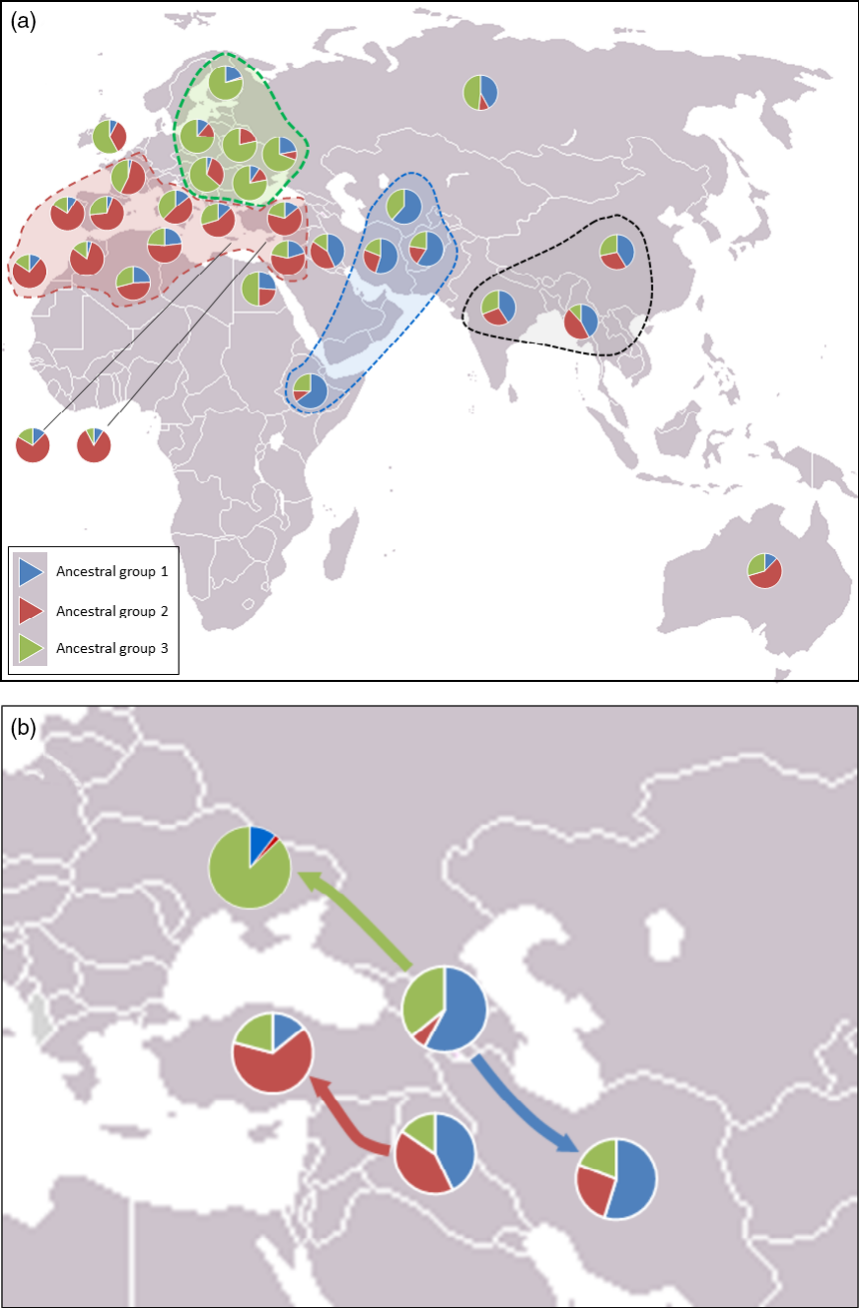
Pie charts for each country from which accessions were collected showing the percentage membership to each of the three groups indicated by STRUCTURE analysis. (a) Western European, North Africa and Australia accessions are dominated by group 2, Eastern European accessions are dominated by group 3, and those from Asian and the Middle East are dominated by group 1. NB Scores for Palestine and Syria have been grouped. (b) The USSR accessions from Caucasian countries (Armenia, Azerbaijan and Georgia) are similar to those from the Middle East and Asia, while those from the Crimea and the Ukraine are most similar to those from Eastern Europe belonging principally to ancestral group 3. The predominance of ancestral group 1 occurs in Iraq and Turkey.

The geographic distribution of the three STRUCTURE groups was investigated by analysing Q scores (percentage apportioning to ancestral groups) for each country and projecting these onto a world map (Fig. 4). This reveals the possible origin of the three ancestral populations predicted by STRUCTURE. There is a Middle Eastern group of countries that was predominantly assigned to group Q1 (blue segments in Fig. 4). The accessions from Ethiopia are most similar to this group. Accessions from Western Europe and North African are predominantly assigned to group Q2 (red segments in Fig. 4), and those from Eastern Europe are predominantly assigned to group Q3 (green segments in Fig. 4). The accessions from the UK, of which there were only three, appear to be most similar to the Eastern European accessions. Accessions from Australia are most similar to those from Western Europe/North Africa. The regional groupings created in this analysis are not sustained in several cases: accessions from Syria, Palestine (grouped into a single pie chart in Fig. 4) and Turkey, on average, are more similar to the Western European and North African accessions than they are to the accessions from other countries in the Middle East. The accessions from Iraq form a ‘*half‐way‐house*’ between these Turkish/Syrian accessions and those from Iran, Afghanistan and Turkmenistan that are predominantly assigned to group Q1. Finally, the samples from the former USSR, a geographically large area, are somewhat similar to both the Asian/Middle Eastern and Eastern European accessions. However, if one considers the country of origin within the former USSR, two groups fall out; accessions collected from the Ukraine and Eastern Russia (Rivers Don and Volga) are most similar to those from Eastern Europe; accessions from the Caucasus are most similar to those from the Middle East and Asia. Of the 26 lines from the former USSR that fell into group Q1, 22 were from Transcaucasia (Armenia, Azerbaijan and Georgia). Of the 19 that fell into group Q3, eleven were from the Ukraine and Siberia. Thus, the lines from the former USSR fall into two groups geographically and are related to either lines in Eastern Europe (group 3) or lines from the Middle East (group 1). Interestingly, along the corridor of land between the Black and Caspian Seas, there is a shift in assignment from predominantly group 3 (the Ukraine) to predominantly group 2 (Palestine/Syria and Turkey) via predominantly group 1 (Transcaucasian countries).

### How distinct is the Watkins Collection from modern, elite varieties?

**Figure 5:**
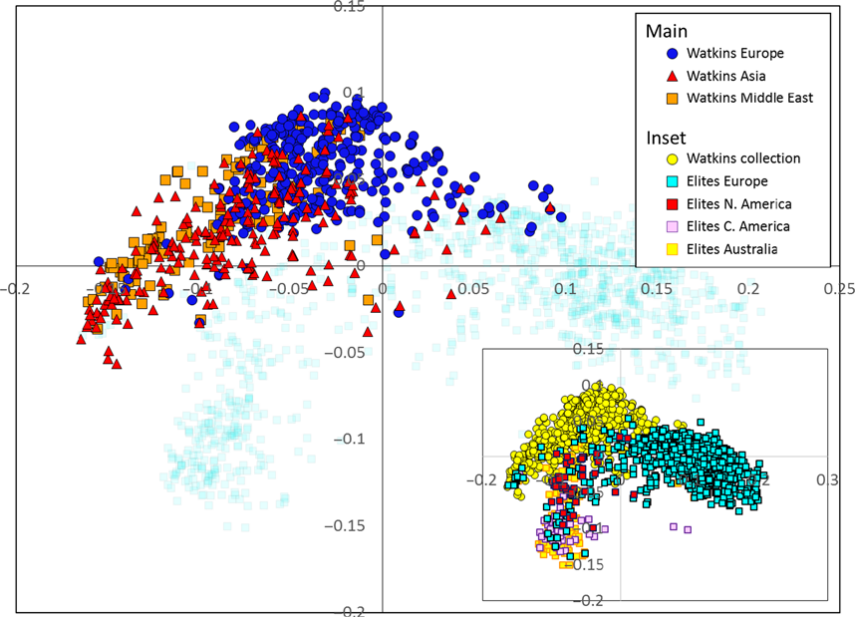
PCO plot (inset) showing the relationship between the accessions belonging to the Watkins Collection (yellow circles) and a collection of elite lines (European = light‐blue squares; N. American = red squares; Australian = yellow squares; C. American = pink squares) analysed on the 35K array. The main diagram is the same plot with the accessions from the Watkins Collection coloured by region of origin; the European accession of the Watkins collection (dark‐blue circles) cluster more closely to the elite European accessions than do those from Asia (red triangles) and the Middle East (orange squares).

To determine whether there is novel variability in the Watkins Collection with respect to modern elite varieties, Watkins accessions were genotyped alongside a large number of elite varieties. An analysis was carried out of 1795 accessions (Watkins = 792, modern elites = 1003) on the 35 k Wheat Breeders’ Array (Allen *et al*., 2016). To visualize the relationship between the individuals in the two collections, a PCO analysis was performed on the joint data set. Three main clusters formed. The modern accessions fell into two clusters; European elites formed a cluster along the positive values of the first principal component while the elite accessions from the Americas and Australia formed a second cluster positioned towards the negative values of both the first and second coordinates (Fig. 5, inset). The Watkins accessions formed a cluster adjacent to, but distinct from, the other two clusters. This latter cluster was orientated such that the Watkins accessions of European origin lay immediately adjacent to the European elites, whereas the Asian and Middle Eastern accessions were most distant from them (Fig. 5).

**Figure 6:**
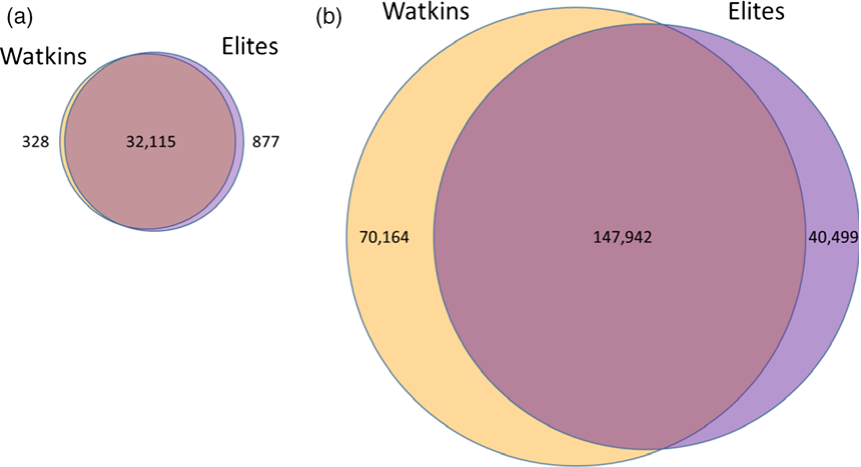
Venn diagrams of the polymorphic SNP markers on the 35 K and 820 K Arrays. (a) On the 35 k array, there are very few polymorphic markers unique to either of the two collections; (b) on the 820 k array, the Watkins and elite collections have a large number of unique polymorphic markers.

Of the 35 143 markers on the 35 k Breeders’ array, 33 320 (94.81%) were polymorphic across the two collections taken together; 32 443 (92.32%) and 32 992 (93.88%) were polymorphic markers in the Watkins Collections and elites, respectively. Thus, the majority of markers (32 115) were polymorphic in both collections, and very few polymorphic markers were exclusive to either group (Fig. 6a); 328 (1.0%) and 877 (2.7%) polymorphic markers unique to the Watkins and elite accessions, respectively.

### Further comparison of the Watkins Collection and modern, elite varieties using the 820 K array

To further investigate the relationship between modern accessions and the Watkins Collection, we characterized the core collection of 120 Watkins accessions and 145 elite cultivars using the wheat high‐density 820 K Axiom Array, which contains 819 571 markers (Winfield *et al*., 2016). Using this array with the above samples revealed 258 605 (31.55% of markers on the array) polymorphic markers (Fig. 6). In the accessions from the Watkins Core Collection, there were 218 106 polymorphic markers of which 70 164 (32.2%) were unique to this collection. In the elite accessions, there were 188 441 polymorphic markers of which 40 499 (21.5%) were exclusive to members of this collection (Fig. 6b and File S3; *Lists of collection specific, polymorphic SNP markers and their chromosome positions*).

### Novel polymorphisms in the Watkins core collection

**Figure 7:**
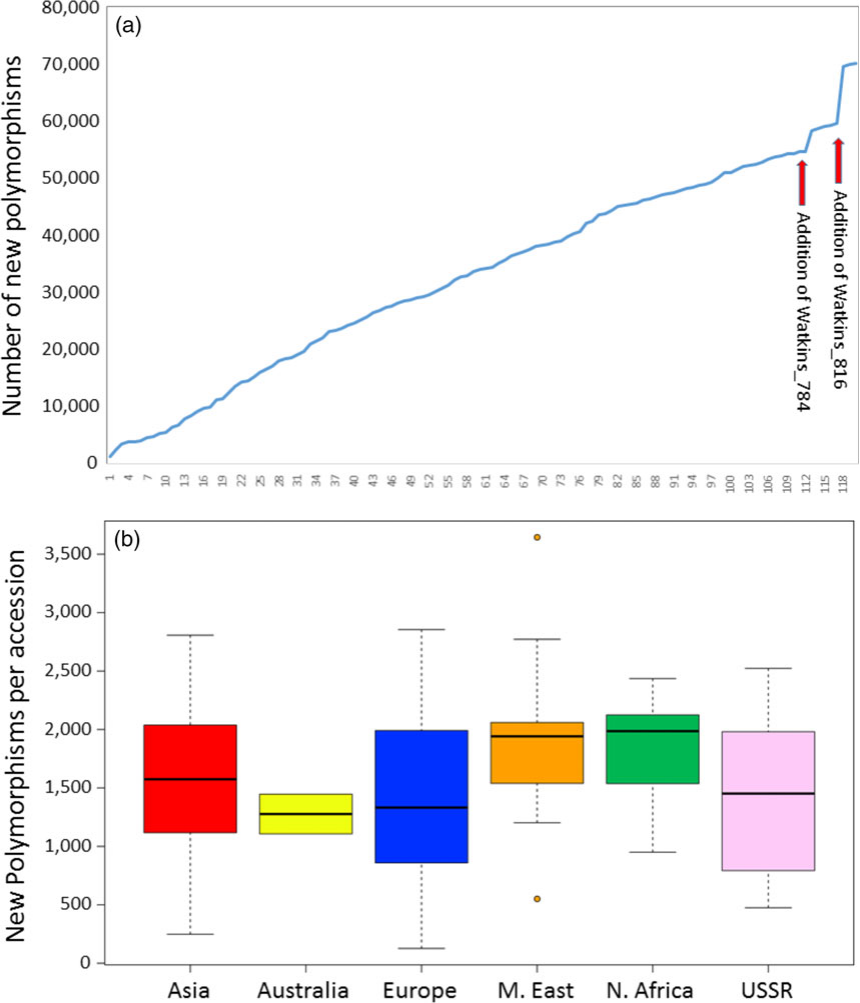
(a) Plot showing the accumulative addition of new polymorphisms as additional Watkins accessions are added to the scores for the elite lines. (b) Box and whisker plots showing the number of additional polymorphic markers introduced as each accession from the different regions was added to the elite accessions in turn. The Middle Eastern plot is flanked by two outliers, one high and one low. The two outlying Italian accessions, Watkins_784 and Watkins_816, are not included in this figure.

Having determined (using the 820 k HD Array) that, with respect to the 145 modern elite accessions used in this study, the Watkins Core Collection contains a considerable number of novel polymorphisms, we wished to determine which of the Watkins accessions were particularly polymorphic and whether there was any geographic bias in this regard. That is, are accessions from some regions more polymorphic than accessions from other regions? To determine this, the genotype scores for each Watkins accession in turn were added to the genotype scores for the 145 elites and the number of additional polymorphisms was determined. The cumulative addition of the Watkins accessions of the Core Collection shows that each contains novel polymorphisms (Fig. 7a). The average number of new polymorphisms per accession was 1662 (range 127–12 828). The largest number of new polymorphisms was present in an accession from Italy (Watkins_816), the smallest from a French accession (Watkins_040). Two accessions, both from Italy (Watkins_784 and Watkins_816), stood out as being highly polymorphic: accession 784 contributed 5677 new polymorphisms and accession 816 added 12 828; these were considered to be outliers. In general, accessions from the Middle East and North Africa contributed the largest number of new polymorphisms, accessions from Australia and Europe (with the two Italian outliers removed) the smallest number (Fig. 7b). Interestingly, there was a distinct difference in the average number of novel polymorphism between the accessions of Eastern and Western Europe. The accessions of Eastern European origin had, on average, many fewer novel polymorphisms (average number = 1067; range 635–2028) compared to those from Western Europe (average number = 1502; range 127–2855). These data are available in File S4, *Novel alleles in each of the Watkins accessions*.

### Chromosome distribution of polymorphic markers

**Figure 8:**
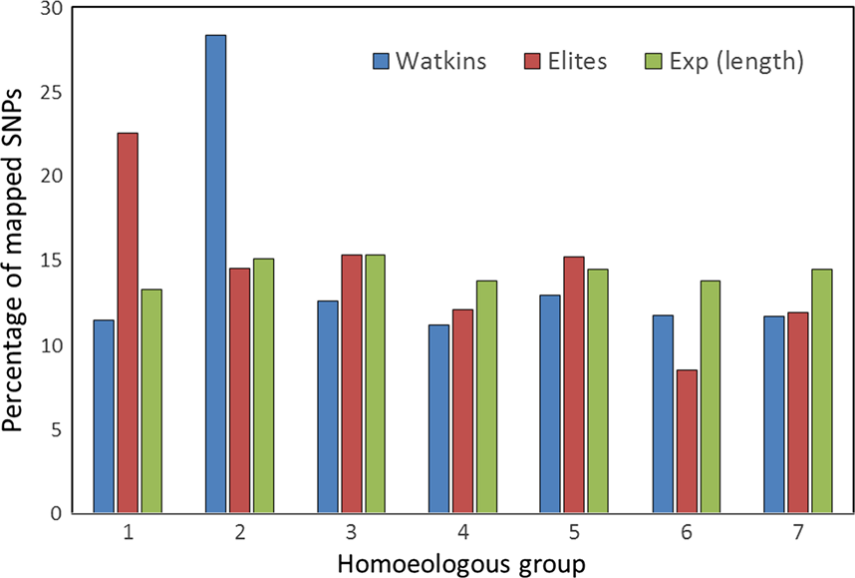
Bar graph showing the percentage of polymorphic markers unique to the two collections (Watkins vs. elites) that mapped (BLASTN to Chapman) to the seven homoeologous groups compared to the expected number based on chromosome size determined by Gill *et al*., 1991. In the Watkins accessions, more markers mapped to homoeologous group 2 chromosomes than would be expected given the physical size of the homoeologous groups; in the elite accessions, more markers mapped to group 1 chromosomes.

Map locations of the 218 106 polymorphic SNP markers identified in the 120 accessions of the Watkins Core Collection were determined by BLASTN (Altschul *et al*., 1990) to the Chapman POPSEQ genome assembly (Chapman *et al*., 2015). Only 127 043 of the polymorphic markers could be assigned a chromosome location; of these, 16 687, 30 859, 18 147, 12 596, 16 637, 15 554 and 16 563 mapped to homoeologous chromosome groups 1–7, respectively. For the elite accessions, only 110 314 of the polymorphic markers could be assigned a chromosome location; of these, 17 607, 22 559, 16 722, 10 959, 15 043, 12 761 and 14 663 mapped to homoeologous chromosome groups 1–7, respectively. Given the relative physical sizes of the seven homoeologous groups (based on the physical dimensions given in Gill *et al*., 1991), this would suggest that more polymorphic markers mapped to homoeologous group 2 chromosomes and slightly less to homoeologous group 4 chromosomes than would be expected (File S3; *Lists of collection specific, polymorphic SNP markers and their chromosome positions*). Of the polymorphic markers unique to the two collections, 42 492 (Watkins) and 25 764 (elites) could be provisionally mapped based by BLASTN to the Chapman assembly (Chapman *et al*., 2015). These markers were not evenly distributed between the seven homoeologous chromosome groups (Fig. 8). That is, assuming proportional distribution of markers to the homoeologous groups based on physical length (using the estimated size of the homoeologous groups of the variety Chinese Spring as a proxy (Gill *et al*., 1991), more of the polymorphic markers than would be expected were found on group 2 chromosomes in the Watkins accessions and in group 1 chromosomes in the elite accessions. The elite accessions also appeared to have fewer than expected number of polymorphic markers on homoeologous group 6 chromosomes.

## Discussion

A previous study by Wingen *et al*. (2014) used 41 microsatellites to characterize the Watkins Collection of landraces. While limited in scope, the study suggested that the Watkins Collection had a significantly higher level of diversity than a collection of modern European, winter bread wheat varieties generated during the period 1945–2000 (Wingen *et al*., 2014). The main objective of this current study was to apply recently developed high density and high throughput SNP‐based genotyping techniques to characterize the Watkins Collection in greater detail and to compare both the wider and Core Collection with a larger number of elite hexaploid lines.

We used the recently developed 35 k Axiom Wheat Breeders’ array (Allen *et al*., 2016) to characterize a large number (804) of accessions from the Watkins Collection. This 384 configuration array contains 35 143 SNPs chosen on the basis of good genome coverage and polymorphic information content (PIC) scores when used to screen a global collection of wheat lines that included a number of Watkins accessions (Allen *et al*., 2016). The majority (92.15%) of markers on this platform proved to be polymorphic against the tested Watkins accessions, and cluster analysis revealed some broad geographic partitioning of the accessions. That is, although there was some overlap between clusters, most European accessions clustered together and away from the majority of the Asian and Middle Eastern accessions. Eastern European samples (Bulgaria, Finland, Hungary, Poland, Romania, Yugoslavia) were shown to be distinct from Western European accessions (Canary Islands, Crete, Cyprus, France, Greece, Italy, Portugal, Spain). In contrast to the results reported by Riaz *et al*. (2016) and Cavanagh *et al*. (2013), we noted that the Eastern European cluster contained most (65.5%) of the winter wheat accessions (File S5). Accessions from Australia and North Africa were similar to those from Western Europe. This pattern is not dissimilar to that reported by Wingen *et al*. (2014) but provides a more detailed picture of the geographic distribution of genetic variability.

The most distinct geographic structuring was among the accessions from the former USSR. That is, they principally fell into one or other of two main clusters according to their geographic location within the former USSR. The accessions from Transcaucasia (Armenia, Azerbaijan, and Georgia) clustered with accessions from the Middle East and Asia, while those from north of the Black Sea (Crimea and the Ukraine) clustered with accessions from Eastern Europe. The accessions from Siberia were not distinctly clustered by PCO plot. This highlights an interesting pattern of diversity around the Black Sea: accessions collected from this relatively small geographic area, that is the two regions from the former USSR (Transcaucasia and the Ukraine) and Turkey, are genetically quite distinct and derived from the three main ancestral groups indicated by the STRUCTURE analysis (Fig. 4b). The region between the Black Sea and the Caspian Sea, or just south of this (Iraq), which is the presumed location of the centre of origin of wheat domestication, appears to be where the populations merge (Fig. 4b). Moving west from Iraq, the accessions from Turkey, North Africa and Western Europe are closely related (predominantly STRUCTURE group Q1); moving to the north and west, the accession from the Ukraine is similar to those in Eastern Europe; moving to the south and east, the accessions from the Transcaucasia are related to those in the Middle East (Iran, Iraq) and Asia (Afghanistan and Turkestan). The patterns indicated by the STRUCTURE analysis fit remarkably well with the pattern of domestication described by Feldman (2001): that is, from its origin of domestication in south eastern Turkey, the crop arrived in Europe via a route to Anatolia, then to Greece and from there extended throughout Europe via two routes; one way proceeded northward through the Balkans to the Danube, a second went across to Italy, France and Spain, finally reaching UK and Scandinavia. In a similar way, wheat spread via Iran to central Asia, reaching China, and via Egypt into Africa. Wheat was introduced to Australia by Spaniards in 1788. This latter fact is reflected in the similarity between Australian and Western European accessions (Figs 2 and 4).

To determine whether the accessions belonging to the Watkins Collection are distinct from modern breeding lines in terms of their SNP diversity, a comparison was made between them using the 35 k Breeders’ Array which has been demonstrated to be effective at screening a wide range of germplasm (Allen *et al*., 2016). PCO analysis (Fig. 5) of the pairwise genotypic similarity scores for accessions from the Watkins Collection (*n* = 804) together with modern elites (*n* = 1002) accessions indicates that the former are different from the latter as there was little overlap between their respective clusters. This lack of co‐localization is, in part, explained by the fact that there is not complete geographical concordance between the countries of origin of the accessions analysed: the elite varieties are principally from the Americas, Australia and Europe while the accessions from the Watkins Collection come from Asia, the Middle East and North Africa as well as from Europe and Australia. However, Watkins accessions of European origin form a cluster distinct from the elite European accessions. Interestingly, the Australian Watkins accessions do not cluster with the elite Australian accessions which might reflect the fact that modern Australian lines are not derived from their local landraces. It has been reported that 98 per cent of the Australian wheat belt is sown with varieties derived from genetic materials from the CIMMYT gene bank and breeding programmes (Braidotti, 2013). In agreement with this, in our study, Australian elite accessions co‐localize almost entirely with the Central American (CIMMYT) accessions. This similarity has also been reported by Riaz *et al*. (2016).

Cluster analysis of the genotype data from the 35 k Breeders’ Array clearly indicated a difference between accessions of the Watkins Collection and modern elite cultivars. However, while the 35 k Breeders’ Array proved efficacious at characterizing a wide range of global wheat accessions by utilizing commonly occurring polymorphisms in these collections (Allen *et al*., 2016), it was not designed to uncover novel variation that one might expect to occur in landrace accessions. The 820 k array, on the other hand, which contains a large number of SNPs generated from a wide range of wheat accessions and species belonging to wheat's secondary and tertiary gene pools (Winfield *et al*., 2016), should be able to uncover a wider range of diversity including any novel alleles only present in one or other of the collections. Indeed, this was the case; using the 820 K array to characterize the 120 accessions of the Watkins Core Collection (described in Wingen *et al*., 2014) alongside 145 elite accessions, identified 258 605 polymorphic markers of which 70 164 (32.17%) were only found in accessions belonging to the Watkins Core Collection and 40 499 which were exclusive to elites (Fig. 6b). Thus, we were able to identify more than 110,000 markers that distinguish Watkins landrace accessions from elite accessions.

The SNPs unique to the landraces might indicate the presence of a valuable genetic variability that is not being incorporated in modern breeding programmes. All 120 accessions of the Watkins Core Collection possessed novel alleles, but there was great variability in the number of these (range 127–12 828) and the accessions from some regions and countries housed more variability than others. At a regional level, the landrace accessions from Australia and Eastern Europe were the least rich with regard to the number of novel polymorphisms. By contrast, it would appear that breeders should turn to landraces in the Middle East and North Africa if they are looking for novel alleles as these, on average, carry the largest number of novel polymorphic alleles. On a country‐by‐country basis, these patterns become a little clearer. The Eastern European landraces did have relatively low numbers of novel SNPs. The landrace accessions from France, however, had by far the smallest number of novel SNPs (*n* = 606). The countries with the largest number, apart from Italy with its two very diverse accessions, were Palestine, Turkestan, Iran and Egypt. It is perhaps no surprise that these SNP rich accessions occupy the ancestral home of bread wheat in the fertile crescent.

### Chromosome distribution of polymorphic markers

Two features stood out with regard to the distribution of the polymorphic markers unique to the two collections. The elite accessions apparently had a greater than expected percentage of polymorphic markers on the homoeologous group 1 chromosomes, while the Watkins accessions had a greater percentage on homoeologous group 2 chromosomes, a pattern that has also been observed by Riaz *et al*. (2016). The large number of unique SNPs on group 1 chromosomes in the elite accessions is probably explained, in part at least, by the 1BS/1RS translocation that is found in many cultivated lines but not in landraces. The larger than expected number of polymorphic markers on the homoeologous group 2 chromosomes in the Watkins accessions was almost exclusively explained by the variability found in a single accession, Watkins_816, collected in Italy. This accession had many polymorphic calls when all other accessions were monomorphic. Further investigation into the nature of this variability, using the Axiom™ CNV Summary Tools Software reported in Allen *et al*. (2016), indicated that Watkins_816 has a whole chromosome deletion for 2D and, in compensation, two copies of chromosome 2A. This analysis also indicated that there might be some translocations on chromosome 2B. The group 2 chromosomes contain several genes of great agronomic importance, including the photoperiod response genes *Ppd1*,* Ppd2* and *Ppd3*, numerous genes conferring resistance to leaf, stem and stripe rust and to powdery mildew, the two semi‐dwarfing genes *Rht7* and *Rht8*, the gametocidal genes *Gc1‐B1a* and *Gc1‐B1b*, and a QTL involved in resistance to preharvest sprouting (Conley *et al*., 2004). Given the importance of the group two chromosomes, the landrace accession Watkins_816 might prove to be of interest to breeders.

Thus, the Watkins Collection would appear to be a valuable reservoir of variability that might be drawn from in future breeding programmes. This array‐based study has provided a clear picture of the level of genetic diversity and its geographic distribution in the Watkins Collection and, in so doing, we hope provides breeders with a useful map to guide them in the choice of material in breeding programmes. In general, it would appear that breeders should turn to landraces in the Middle East and North Africa if they are looking for novel alleles as these, on average, carry the largest number of novel polymorphic alleles. However, there are accessions from all six regions that carry a large number of novel polymorphisms. Thus, array‐based genotyping studies of landraces collections, such as the Watkins Collection, could prove to be highly valuable to breeders allowing them to precisely target the regional alleles they wish to include in their breeding programmes. The diversity present within the Watkins Collection has been captured in an elite background through the large‐scale development of bi‐parental populations and near isogenic lines (NILs) undertaken in the Landrace Pillar of the Wheat Improvement Strategic Programme (WISP). This material has been developed and is free to access in the form of a breeder's toolkit (http://wisplandracepillar.jic.ac.uk/).

## Experimental procedures

### Plant material

Plants for this study (listed in File S1) were obtained from two sources: 804 accessions of hexaploid wheat developed from single seed descent from the Watkins Collection, a collection of wheat landraces made by A.E. Watkins in the 1920s and 1930s from a wide geographic distribution (Fig. 1 and Table 1); 1003 modern, elite, hexaploid bread wheat accessions originating from 17 countries in Africa, Australia, the Americas, the Middle East and Europe.

Plants grown for DNA extraction were potted in a peat‐based soil and maintained in a glasshouse at 15–25°C with 16‐h light, 8‐h dark. Leaf tissue was harvested from 6‐week‐old plants, immediately frozen in liquid nitrogen and stored at −20°C prior to nucleic acid extraction. Genomic DNA was isolated using a phenol‐chloroform extraction method (Sambrook *et al*., 1989), treated with RNase‐A (New England Biolabs UK Ltd. Hitchin, UK), according to the manufacturer's instructions and purified using the QiaQuick PCR purification kit (QIAGEN Ltd., Manchester, UK).

### Axiom array genotyping

Two different Axiom^®^ Array platforms were used in this study: (i) the Axiom^®^ Wheat HD Genotyping Array, which contains 820 k features (Winfield *et al*., 2016) and (ii) the Axiom^®^ Wheat Genotyping Breeders’ Array, which contains 35 k features (Allen *et al*., 2016). The 35 k Wheat Breeders’ Array was used to genotype the 804 accessions of the Watkins Collection alongside 1003 modern elite cultivars. The 820 k Wheat HD Array was used to genotype all 120 accessions of the Watkins Core Collection (Wingen *et al*., 2014) and a set of modern elite cultivars (*n* = 145). Samples were genotyped using the Affymetrix GeneTitan^®^ system according to the procedure described by Affymetrix (Axiom^®^ 2.0 Assay Manual Workflow User Guide Rev3). Allele calling was carried out using the Affymetrix proprietary software package GTC, following the Axiom^®^ Best Practices Genotyping Workflow (http://tools.thermofisher.com/content/sfs/manuals/axiom_genotyping_solution_analysis_guide.pdf).

### Data analysis

#### Dimensionality reduction

The relationship between the lines was determined by calculating a pairwise similarity matrix for all lines using a custom python script. Similarity was calculated as the number of calls in common between two lines divided by total number of markers scored for the two lines; markers that had missing calls for either of the lines being compared were not used to estimate similarity. The matrix was imported into the R statistical software package version 3.3.1 (R Core Team, 2013), and principal co‐ordinates (PCO) were calculated using the classical multidimensional scaling (MDS) function, ‘*cmdscale*’. The first two principal co‐ordinates were plotted.

### Statistical analysis

All descriptive statistics were calculated using the R software package version 3.3.1 (R Core Team, 2013).

### Structure analysis

To investigate population structure for the Watkins Collection (*n* = 804), the Bayesian model‐based clustering method implemented in the program STRUCTURE version 2.3.4 (Pritchard *et al*., 2000) was used. Setting used were as follows: admixture, burn‐in of 10 000, reps 10 000. The number of populations assumed was between one and twenty with five replicate runs for each assumed population size. The best fit for the number of clusters, K, was determined using the Evanno method (Evanno *et al*., 2005) as implemented in the program STRUCTURE HARVESTER (Earl and von Holdt, 2012). The results from STRUCTURE were submitted to CLUMPAK version 1.1.2 (Kopelman *et al*., 2015) in order to align cluster assignments across replicate analyses and produce visual representations of the cluster assignments.

## Supporting information


**File S1** Accessions used in this study.

**File S2** Principal co‐ordinate plots of Watkins accessions coloured by country of origin.

**File S3** Markers that are polymorphic only among Watkins accessions or elite accessions.

**File S4** Number of novel SNP polymorphisms in each Watkins accession.

**File S5** Principal co‐ordinate plot of Watkins accessions coloured by sowing season.
